# Effect of Dry–Wet Cycling on Methanotrophs in Wetland Soils

**DOI:** 10.3390/biology15030279

**Published:** 2026-02-04

**Authors:** Xi Zhu, Zhihao Zhang, Anan Du, Bingru Liu

**Affiliations:** 1 College of Biological Science and Engineering, North Minzu University, Yinchuan 750021, China; 2 Ningxia Baijitan Forest Ecosystem Observation and Research Station, Lingwu 750499, China

**Keywords:** wetland soil, dry-wet cycling, soil methanotrophs, methane emissions

## Abstract

Wetlands occupy a mere 6% of Earth’s land surface, yet they contribute 25–45% of global natural methane (CH_4_) emissions. A key contradiction emerges here: it is the soil of these wetlands that serves as the host for methane-trophic microorganisms, which can oxidize the vast majority of the methane they produce under specific conditions (for example, the aerobic interface). A wetland’s role as either a net source or sink for atmospheric CH_4_ is therefore a primary driver of hydrological variability. This research synthesis current understandings of how wet–dry cycles regulate methanotrophic communities and their CH_4_ consumption capacity. Shifts in the water table directly modulate methanotroph physiology, community structure, and metabolic activity. These hydrological effects are further amplified or attenuated by nitrogen availability, plant-derived exudates, and edaphic properties. Herein, key knowledge gaps concerning the adaptive responses of methanotrophs to hydrological change are identified, and targeted research priorities improving predictions of wetland CH_4_ fluxes under contrasting moisture regimes are accordingly outlined. This review synthesizes recent advances to highlight the mechanistic understanding essential for guiding wetland management strategies.

## Simple Summary

Wetlands are a major source of methane emissions, and their hydrological cycles play a crucial role in regulating methane release through their influence on soil microorganisms. This review examines how hydrological rhythms affect the community structure and function of methanogens and methanotrophs in wetlands, while also considering the roles of plants, soil properties, and other influencing factors. Based on this analysis, we propose future research directions and potential control strategies for wetland methane emissions. These suggestions will contribute to improving the prediction of methane emissions from wetlands under climate change and provide a scientific basis for wetland management.

## 1. Introduction

Methane (CH_4_) represents one of the most important greenhouse gases in the atmosphere. Despite its low concentration, the contribution of CH_4_ to the greenhouse effect is 28–34 times that of the same mass of carbon dioxide (CO_2_). Meanwhile, the average retention time of methane in the atmosphere can reach 9–12 years, which is much longer than the turnover time of carbon dioxide, which is on average 4 years [1]. This paper selects the common wetland systems in the research and explores the impact of dry and wet altercation of wetlands on methane emissions by comparing natural wetlands (peatlands, temperate wetlands, tropical wetlands, and permafrost zones at the North and South Poles) with artificial wetlands (rice fields and slightly lower zones of reservoirs). Wetlands, covering 6–8% of the total land area, store 18% of the total terrestrial carbon and account for approximately 35% of the total global CH_4_ emissions [2,3]. Therefore, CH_4_ has emerged as a research focus in terms of global warming. Anaerobic conditions in wetland soils position wetland ecosystems as a major source of CH_4_; however, not all CH_4_ produced by wetland ecosystems is released into the atmosphere. Approximately 60–90% of wetland-derived CH_4_ is oxidized by soil methanotrophs prior to its release into the atmosphere [4]. Therefore, soil methanotrophs serve as key regulators of the CH_4_ released from the biosphere to the atmosphere, playing a crucial role in maintaining the Earth’s atmospheric CH_4_ balance.

Under periodic dry–wet cycling, the duration of flooding and the water level are the main factors affecting methanotrophs in wetland soils. The periodic flooding–drying process results in dramatic changes in the physicochemical and biological properties of wetland soils, thereby affecting the dynamics of the production, oxidation, transportation, and final release of CH_4_ into the atmosphere. This is a blind spot in CH_4_ emission control and has become a central research focus [5,6,7]. Climate change amplifies dry–wet cycling frequency, with over 30% of global wetlands experiencing altered hydroperiods [8]. Such fluctuations generate oscillatory selective pressures: aerobic methanotrophs endure desiccation stress during drought periods, whereas anaerobic lineages undergo competition with methanogens under inundated conditions [8]. Comprehending niche partitioning under hydrological stress is vital for anticipating CH_4_-climate feedback.

This review aims to systematically examine the mechanisms by which drying–rewetting cycles affect the community structure and functional activity of methanogens, focuses on static hydrological conditions of different areas. The novelty of this paper lies in its focus on the critical driving factor, hydrological dynamic change, and attempts to integrate this across different perspectives of microbial ecology and biogeochemical processes.

## 2. Hydrological Controls on CH_4_ Release and Oxidation Pathways

Recent years have seen a surge in CH_4_ content stemming from diverse sources; notably, 60% of this increase arises from the simultaneous expansion of wetlands, ruminant stocks, and oil and gas operations. Regarding the surge in wetland emissions, their main controlling factor is wetland area expansion, which is induced by elevated tropical temperatures and enhanced regional precipitation [9]. CH_4_ emission sources primarily include anthropogenic sources and natural sources, among which the wetland ecosystem is the largest natural source of CH_4_ emission [10]. CH_4_ release and oxidation processes in wetland soils significantly influence the level of CH_4_ present in the atmosphere.

### 2.1. Release and Oxidation of CH_4_ in Wetland Soils

The status of a wetland as a net source or sink of atmospheric methane is determined by the balance between its microbial communities. If the abundance and activity of methanogens surpass those of methane-oxidizing bacteria (*methanotrophs*), the wetland becomes a net source of CH_4_ emissions. Otherwise, it functions as a methane sink. In essence, the global methane flux from wetlands hinges on the equilibrium between microbial methanogenesis and methanotrophy. Consequently, the abundant methanotroph communities in wetland soils play a vital role in modulating this critical atmospheric balance. The alternation between drying and wetting cycles in wetlands dynamically shifts soil conditions between anoxia and oxygenation, directly influencing the relative abundance of methanogenic and methanotrophic communities [11]. Notably, members of the *Methylocystaceae* family are key methanotrophs responsible for methane oxidation during these transitional periods. Their global prevalence and broad adaptability are evidenced by their detection across soil environments on all six continents, as revealed through analysis of publicly available 16S rRNA and *pmoA* gene amplicon datasets [12]. Consequently, understanding the dynamics of methanotroph communities, such as those of *Methylocystaceae*, is a central focus in research aimed at predicting and managing wetland methane emissions.

### 2.2. From Tropics to Poles: CH_4_ Responses to Altered Hydrological Rhythms

Natural wetlands typically exhibit substantial spatial heterogeneity in the water level, soil quality, and vegetation. Frequent dry–wet cycling alters soil physicochemical properties and above-ground vegetation, which in turn drives shifts in the community structure and activity of methanotrophs [13,14,15]. Wetland hydrological fluctuation patterns, which are modulated by a range of hydro-meteorological and geological factors, allow the characterization of their hydrological status via parameters of amplitude and period. As shown in Table 1, this section reviews the varying hydrological regimes in floodplains, mires, tundra, rice paddies, and reservoir drawdown zones, examining their impacts on methane emissions and uptake in wetlands from the perspectives of both natural dynamics and human regulation.

In floodplain wetlands, rivers and rainfall bring about long-term and high water level changes. For instance, the water level of the Yangtze River rises by 0.8 m [16], and in the Amazon River [17], it can even exceed several meters. Such hydrological changes have accordingly altered the methane-oxidizing bacterial community. Aerobic conditions during the drying period inhibit the methanogenic process and promote CH_4_ oxidation, significantly decreasing CH_4_ flux throughout the hydrological cycle (such as 32%). However, CO_2_ emissions may increase, leading to changes in the greenhouse gas balance. In peat marshes, a drop in water level typically reduces the CH_4_ flux by 30–46%. In northern peatlands, moderate dryness and wetness may stimulate the oxidation of CH_4_ and turn it into a “sink” of CH_4_ [18]. Within the polar tundra (Arctic and Antarctic), water level variations are comparatively small (e.g., around 5 cm), driven by the seasonal cycle of thawing and freezing, alongside summer rainfall inputs [18]. Temperature serves as a stronger dominant factor compared to hydrology. Against the background of warming, deeper and faster thawing will remarkably stimulate CH_4_ emissions [19].

As artificially managed wetlands, paddy fields exhibit a distinct, short-cycle dry–wet alternation, with the cycle typically spanning 7 to 15 days. Mid-term sun-drying (5 to 10 days) can reduce CH_4_ emissions throughout the growing season by approximately 26% (meta-analysis) [20], which is a verified emission reduction strategy. The fluctuation zone of reservoirs is the hotspot of CH_4_ emission. Due to its great variability in water level (the daily dispatch of tropical reservoirs is 1 m, and the annual variation in the Three Gorges is 30 m), a strong “pump” effect is formed, and its flux is 2.3 times higher than that of the natural flood plain [21,22,23]. Overall, lowered water levels enhance redox potential, a change that restricts methanogen proliferation (especially the acetotrophic Methanosaetaceae) and alters their community structure, leading to decreased CH_4_ emissions [24,25].

### 2.3. Seasonal vs. Event-Scale Hydrological Pulses

Wetland hydrological conditions vary significantly with seasons. Influenced by climate change, the hydrological cycle in tropical wetlands has intensified, with seasonal rainfall expanding wetland areas [26,27,28]. However, when rising temperatures lead to evaporation exceeding precipitation, wetlands may shrink. Abnormal climate change may continuously alter regular drought and precipitation patterns, leading to increased frequency and intensity of alternating dry–wet conditions of the soil [29]. Seasonal variations often influence the spatial distribution of methanotrophic bacterial communities through changes in water levels, thereby affecting their methane oxidation rates. Whereas seasonal wet–dry cycles are the main regulatory driver in natural wetlands, human management is the most significant factor in constructed wetlands, with seasonal changes serving primarily a buffering and auxiliary function.

Short-term seasonal hydrological pulses play a critical role in modulating CH_4_ emissions from wetlands. In spring, snowmelt causes a rapid rise in water levels in temperate wetlands, sharply reducing redox potential and driving a 1–2 order of magnitude increase in methanogen abundance within days, resulting in a 3–5 fold surge in CH_4_ flux [30]. In summer, tropical mangroves are influenced by evaporation and typhoons. Diurnal water-level fluctuations and storm-induced pulses can quickly activate methanogenic genes; yet they only trigger brief, small emission peaks [31]. In the dry subsite of the wetland, more stagnant hydrological conditions during summer and autumn promoted the dominance of type II methanotrophs over type I methanotrophs, as evidenced by both community composition and pmoA gene transcript profiles [32]. In autumn, managed water drawdown leads to a shift in the dominant methanogens from Methanothrix to Methanobacterium, reducing CH_4_ flux by approximately 40% [16]. In winter, freeze–thaw cycles of ice cover in Arctic tundra can activate dormant microbial communities within hours after thawing, elevating emissions from near zero to detectable levels [19]. The study by Chowdhury TR et al. revealed that Type I and Type II methanotrophs dominated the microbial communities in the pulsed and permanently flooded zones in winter [33]. These processes collectively demonstrate that hydrological rhythms finely regulate CH_4_ release from wetlands by controlling microbial activity and community structure.

## 3. Plant–Soil–Nitrogen Cascades Regulating CH_4_ Cycling

### 3.1. Effects of Vegetation Cover on CH_4_ Emissions from Wetlands

**Figure 1:**
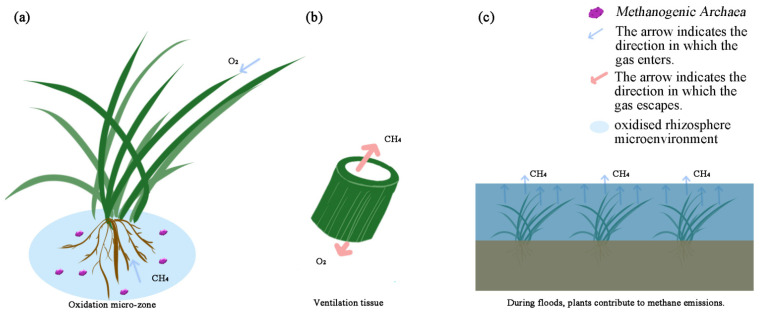
The dual role of plants in methane dynamics. (**a**) Rhizospheric Oxidation: Oxygen released from roots forms an oxidized microzone, where methane is oxidized to carbon dioxide. (**b**) Vascular Transport: Methane diffuses into roots and is transported upwards via the vascular system through specialized aerenchyma. (**c**) Structural Emission: During flooding, plants become the dominant conduit, releasing methane directly into the atmosphere through above-ground structures such as stomata or lenticels.

Vegetation, through its physical structure (coverage) and physiological functions (vascular/aeration), operates as a “bidirectional regulator” in dry–wet alternation. During flooding, it can act as both an “emission accelerator and buffer,” whereas in dry periods, it converts to an “emission inhibitor.” As shown in Figure 1, plants play a dual role in the methane emission process of wetlands. The overall effect depends on the matching interplay between vegetation type, cover density, and hydrological stage.

#### 3.1.1. Rhizosphere Oxidation: The Plant-Mediated CH_4_ Sink

Wetland plants significantly enhance CH_4_ emissions through their well-developed vascular systems. Continuous pathways with low resistance are formed by aerenchyma and vascular bundles, enabling the rapid diffusion of dissolved CH_4_ from anaerobic soils into the atmosphere [34]. Concurrently, oxygen leakage from roots creates an oxidized rhizosphere microenvironment, where methanotrophs oxidize a substantial portion of CH_4_ to CO_2_ before it escapes [35]. The efficiency of this plant-mediated transport is regulated by interspecific variation in aerenchyma formation, plant phenology, and root exudate-driven microbial activity [36,37]. However, current biogeochemical models often oversimplify these processes as static plant transport coefficients, neglecting dynamic plant–microbe interactions and diurnal physiological controls [38,39]. Future models should thus integrate meta-omics insights with mechanistic descriptions of gas transport to more accurately project wetland CH_4_ fluxes under changing climates.

#### 3.1.2. Vascular Transport: The Plant-Mediated CH_4_ Conduit

Wetland plant stems are equipped with aerenchyma, a specialized tissue distinguished by a system of interconnected lacunae. These tissues form low-resistance vascular channels with high diffusion coefficients, allowing CH_4_ to be transported directly from soil pores to the atmosphere and thus bypassing the surface oxidation zone. This process, known as “plant-mediated transport,” is far more efficient than diffusion or ebullition (bubble transport) [39]. The rate of this transport is influenced by plant species, stem diameter, and temperature. In plants such as tropical mangroves and common reeds (*Phragmites australis*), the transport coefficient can reach 0.3–0.6 cm h^−1^, effectively creating a “highway” for CH_4_ emissions. Experimental studies have shown that cutting off these aerenchyma tissues results in a 40–50% decrease in CH_4_ flux [40].

#### 3.1.3. Net Regulatory Effect: Balancing Oxidation and Transport

Vegetation mediates the impact of water-level fluctuations on CH_4_ emissions through a dynamic interplay between its physical structure and physiological functions. During inundation, well-developed aerenchyma acts as a chimney, facilitating CH_4_ escape, while radial oxygen loss creates an oxidative rhizosphere that mitigates a portion of the flux. Upon drawdown, the cessation of carbon exudation and the persistence of oxidized rhizosphere conditions synergistically suppress methanogenesis. The most critical interaction occurs during re-flooding: damaged roots release a pulse of labile carbon that quickly activates methanogens, yet the restoration of oxygen transport mediated by plants lags behind. Such temporal asynchrony between microbial response and plant regulatory function is a key factor underlying the characteristic post-rewetting CH_4_ emission peak observed in many wetland systems.

### 3.2. Influence of Nitrogen on CH_4_ Emissions from Wetlands

Nitrate exerts a potent suppressive effect on wetland CH_4_ emissions, primarily through its role as a preferred alternative electron acceptor in anaerobic respiration [41,42]. On the one hand, in anaerobic soil, denitrification reactions are superior to carbon dioxide reduction, inhibiting the CH_4_ production process [21]. Applying nitrogen fertilizer to paddy field soil can reduce the methane production potential by 35% [43]. On the other hand, nitrite (NO_2_^−^) and nitrogen oxides (NO) are produced during the denitrification process, and these compounds can directly inhibit methanogen activity [44]. In addition, in oxic–anoxic interfaces, the presence of nitrate nitrogen promotes the co-metabolic activity of some CH_4_-oxidizing bacteria and enhances the oxidative oxidation of CH_4_. Consequently, NO_3_^−^ input typically leads to a significant net reduction in CH_4_ flux from wetlands, establishing it as a key factor in managing greenhouse gas emissions from fertilized ecosystems.

NH_4_^+^ exhibits a more complex influence on the release of CH_4_ from wetlands, achieving a balance through the interaction of its direct inhibition and indirect stimulation. Direct inhibition mainly occurs through two pathways: Firstly, it competes with methanotrophs for the active site of methane monooxygenase (MMO)—a key enzyme responsible for CH_4_ oxidation, thereby damaging this crucial process. Secondly, high levels of NH_4_^+^ exhibit direct toxicity to microorganisms, disrupting the cellular homeostasis of the community [45]. However, low doses of ammonium nitrogen can also act as a nutrient to stimulate the overall activity of plants and microorganisms, consequently promoting CH_4_ emissions. Complementarily, NH_4_^+^ also stimulates plant growth, increasing root exudates that provide the necessary substrates for methanogenesis. This indirect promotion often outweighs direct inhibition in productive wetlands and thus leads to a net increase in emissions—a phenomenon termed the “field paradox” [46]. Therefore, the net impact of NH_4_^+^ is highly dependent on both its dosage and the specific wetland system. Low-to-moderate doses typically promote CH_4_ emissions in productive wetlands, while high doses or application in systems with restricted plant growth may lead to net inhibition.

### 3.3. Key Soil Drivers of CH_4_ Emissions in Wetlands

Beyond water level, inherent soil properties modulate the vegetation–nitrogen signal on methanogens. The primary driving forces behind shifts in methanotroph community structure vary across different habitats. For example, soil C/N, organic carbon, total nitrogen, and soil water content are main driving forces for the community structure differentiation of soil methanotrophs in wetlands with different water contents [25], while soil pH or available water and water pH are key environmental factors affecting the community structure and diversity of methanotrophs in grasslands. However, one study reported no significant correlations between the abundance of methanotrophic bacteria in dry land soil and climate or soil properties, and the community structure of methanotrophs was found to be highly correlated with soil pH, organic carbon, annual average temperature, seasonality precipitation, and sand content [47]. Another study revealed that methanotroph distribution showed a significant correlation with soil water content, nutrients, and metal ions, further suggesting that habitat factors play a critical role in shaping the distribution of methanotrophic community diversity [48].

Soil properties regulate CH_4_ emissions through a hierarchy of controls that govern microbial metabolism. At the foundational level, soil redox potential (Eh) and temperature function as master switches. With the increase of Eh, methane emissions also increase [49]. The concentration and lability of soil organic carbon, particularly dissolved fractions, provide the ultimate substrate for methanogenic archaea. Concurrently, the availability of nitrogen and competitive electron acceptors (e.g., Fe^3+^, SO_4_^2−^) modulates the flow of electrons, potentially diverting them away from methanogenesis. Finally, conditioning factors such as pH and texture exert modulating effects. pH exerts a formative influence on methanogen community composition [50], favoring different pathways (acetoclastic vs. hydrogenotrophic). When the C/N ratio exceeds 20, the growth of acetoclastic Methanothrix is favored, whereas a ratio below 12 enhances methylotrophic oxidation [51]. Furthermore, clay content can protect organic matter from decomposition, facilitating the accumulation of a long-term carbon bank.

The integrated effect of these drivers is nonlinear and context-dependent. For instance, the stimulatory effect of warm temperature on methanogenesis is fully expressed exclusively under conditions where Eh is adequately low and labile carbon is accessible. Statistical models across wetland types consistently identify the combination of Eh (or water table depth as its proxy) and temperature as the primary drivers of spatial and temporal flux variability, with substrate availability modifying the magnitude of this response. Therefore, predicting CH_4_ emissions requires models that capture not just individual factors but also their hierarchical interactions within the soil matrix [52,53].

When the habitat is stressed by a disturbance, methanotrophic communities show varying levels of tolerance to the stress, resulting in changes in the methanotrophic community diversity, which in turn affects the rate of CH_4_ oxidation and has important impacts on CH_4_ balance [54,55].

### 3.4. Summary and Perspectives

This chapter demonstrates that wetland CH_4_ emissions are governed by an integrated plant–soil–nitrogen cascade. Vegetation actively regulates the net flux by balancing rhizosphere oxidation (a CH_4_ sink) against vascular transport (a CH_4_ conduit), with the outcome shaped by plant traits and local hydrology. Nitrogen input exerts form-dependent effects: nitrate strongly suppresses methanogenesis, while ammonium and organic nitrogen can have dual, context-dependent roles influenced by dose and soil conditions. Fundamentally, soil properties impose ultimate constraints on these biotic and nutrient-related processes. Redox potential and temperature function as master switches in this context, while the availability of organic matter determines the overall magnitude of such processes. In essence, CH_4_ flux emerges not from any single factor, but from the hierarchical interaction where plants influence soil nitrogen dynamics, and both operate within the physicochemical limits set by the soil matrix. In this case, models that can capture these cascading interactions are required to predict emissions.

## 4. Methodological Advances in Linking Community Dynamics to CH_4_ Fluxes

### 4.1. Dry–Wet Cycling Altering the Characteristics of Methanotrophs

Studies have demonstrated that dry–wet cycling remarkably affects the physiological properties, community structure, and functions of methanotrophs. For instance, during the dry phase, increased oxygen penetration (0–10 cm depth) stimulates the activity of aerobic methanotrophs (e.g., *Methylococcus*, *Methylocystis*), whereas prolonged desiccation induces oxidative stress [56]. During the wet phase, rapid oxygen depletion reactivates anaerobic methanogenesis, while anaerobic methanotrophs (e.g., ANME-2d) couple CH_4_ oxidation to NO_3_^−^/Fe^3+^ reduction [57]. In addition, dry–wet cycling can further change the metabolic activities and ecological niches of methanotrophs by affecting factors such as substrate availability, pH, and ion concentrations.

Drying reduces CH_4_ solubility, limiting diffusion to methanotrophs. Post-rewetting, trapped CH_4_ releases as abrupt pulses [58]. Drought accelerates SOM mineralization, increasing labile carbon for methanogens upon rewetting. Evaporation during drying concentrates salts (e.g., SO_4_^2−^, Cl^−^), inhibiting methanotroph enzymes (e.g., particulate methane monooxygenase, pMMO). Rewetting dilutes salts but may trigger osmotic shock [9]. As a result, the wetland hydrological changes of community composition of methanogens have a significant impact, in turn affecting the wetland methane emissions.

*Methanotrophs* are extensively distributed in the Earth’s ecosystem and are classified as γ-Proteobacteria (type Ia/Ib), α-Proteobacteria (type II), and Verrucomicrobia (those that grow only in extreme environments). Type I and type II methanotrophs are categorized based on their physiological and ecological differences, including differences in membrane structure, carbon assimilation patterns, and membrane phospholipid fatty acids, widely present in various ecosystems [59].

It has long been hypothesized that type I methanotrophs dominate oxic, low-CH_4_ habitats, whereas type II methanotrophs prefer anoxic or micro-oxic zones with elevated CH_4_ [60]. Some studies have verified that type II methanotrophs are more tolerant to anaerobic habitats, while type I methanotrophs prefer well-aerated habitats [61].

**Figure 2:**
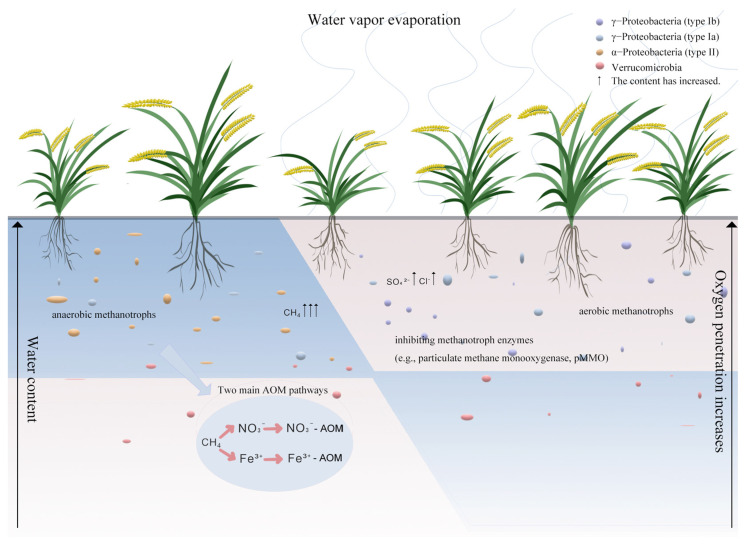
Effect of dry and wet alternations on CH_4_-oxidizing bacteria. Schematic illustration of methanotrophic and methanogenic community dynamics in paddy wetland soils under alternating wet–dry moisture conditions, highlighting four major groups: γ-Proteobacteria (type Ib) (purple), γ-Proteobacteria (type Ia) (blue), α-Proteobacteria (type II) (yellow), and Verrucomicrobia (red).

Nevertheless, in high-CH_4_ wetlands, type I *methanotrophs* often outnumber type II cells if sufficient O_2_ is supplied by plant aerenchyma or fluctuating water tables [62]. Conversely, in oligotrophic forest soils with only trace CH_4_, USCα—a drought-adapted clade within type II—can account for >70% of pmoA transcripts [63]. Moreover, different species within the type II methanotroph group exhibit dominance across a range of water regime conditions. For example, there is strong competition for substrates in the soils with low moisture content such as in dry land and woodland soils, and USCα (type II methanotrophs that grow in dry land and cannot be cultivated in the laboratory) are the dominant species; type II methanotrophs (*Methylocystis/Methylosinus*) thrive in high-moisture soils—such as swamps and swampy soils—characterized by persistent wetness and high organic matter content [64]. As shown in Figure 2, these studies show that CH_4_ concentration and oxygen availability jointly shape the broad distribution of type I and II methanotrophs, while soil moisture partitions the type II guild into USCα (dry habitats) and *Methylocystis/Methylosinus* (wet habitats) functional subgroups, reconciling the apparent field-scale contradictions.

These results indicate that different species of methanotrophs have varying preferences regarding concentrations of CH_4_ or oxygen in their habitats, resulting in the predominance of one type of methanotroph in a habitat depending on the concentrations of CH_4_ or oxygen.

The methanotrophic community in wetlands is primarily composed of type I and type II methanotrophs [53,64]. However, studies regarding changes in the community structure of methanotrophs in soils with different humidity levels have not drawn consistent conclusions. During alternating dry–wet cycles, the rising water level reduces the soil oxygen concentration, thereby decreasing the amount of available oxygen for methanotrophs; drainage increases the soil oxygen content and promotes the growth of methanotrophs. In submerged soil, the oxygen concentration is lower, a phenomenon driven by the relatively slow diffusion of oxygen through water [60]. The diffusion rate of oxygen in air is approximately 10^3^–10^4^ times faster than that in water. Oxygen in submerged soil is quickly consumed via the respiratory activities of soil microorganisms and plant roots. By contrast, drained soils maintain higher oxygen concentrations, as methanotrophs residing on the soil surface can utilize atmospheric oxygen.

Studies have shown that methanotrophs living at the water–soil (or sediment) interface [65] and in well-aerated zones possess the highest activity, while the methanotrophs living in anaerobic [66] or persistently dry habitats [9] may survive adverse environmental conditions in the form of a “microbial seed bank.” In natural wetland and lake systems, type I or type II methanotrophs, or both, play a role in CH_4_ oxidation [67]. Meanwhile, in disturbed habitats, populations of type II methanotrophs exhibit a faster recovery rate compared to those of type I methanotrophs. Despite a decrease in methanotrophic diversity after a disturbance, methanotrophic activity is not only unaffected compared with undisturbed communities but is also rapidly activated and even overcompensated during restoration [61]. This indicates that the preference and tolerance for different habitats may affect the niche differentiation or activity of methanotrophic communities [14,15]. As shown in Table 2, the adaptation ranges of different methane-oxidizing bacterial communities vary, and different methanotroph types differ in their contributions to the overall CH_4_ oxidation rate.

### 4.2. Changes in the Characteristics of Methanotrophic Species Under Dry–Wet Cycling

In highly disturbed habitats such as wetlands with alternating dry–wet conditions, two key aspects remain unclear: the resource allocation patterns among distinct methanotroph types, and the relationship between CH_4_ oxidation rates and the abundance of different methanotroph groups. Although existing research shows that the flood duration is the key factor of the oxidation of methane bacteria community structure differentiation, flooding duration in riparian zones or lakeshore zones significantly affects the community structure of methanotrophs, indicating that different types of methanotrophic communities adapt to varying flooding gradients through niche differentiation, or that different types of methanotrophs jointly become dominant species in a certain habitat [70]. However, other studies have revealed that the spatial distribution patterns of different types of methanotrophs do not align with variations in flooding duration [71].

Furthermore, research shows that the most abundant ASV (e.g., ASV1, type Ib methanotroph), with a significantly higher relative abundance during the wet period than during the dry period (1.49% vs. 0.69%, respectively), was most closely related to Methylocaldum gracile Kamal Bhat 321. The copy number of the pmoA gene and the abundance and diversity of methane-oxidizing bacteria in the wet period were significantly higher than those in the dry period [12]. The methane-oxidizing bacteria community showed more drift contributions in the wet period, and the methane-trophic community exhibited different environmental preferences/vulnerabilities in the dry and wet periods.

Some researchers have used background phospholipid fatty acid analysis coupled with ^13^C tracers (^13^C PLFA) to delve into the methanotrophic community. Their results have revealed that type I methanotrophs are active in wetlands with different flooding gradients and maintain dominance in such environments. In contrast, type II methanotrophs exist only in continuously submerged wetlands, and their activity levels remain low [12]. However, Siljanen et al. [32] found that in the flooded soil of a wetland, the abundance of type I methanotrophs had a significant positive correlation with CH_4_ oxidation potential, while in the soil of drained wetlands, the abundance of type II methanotrophs was positively correlated with CH_4_ oxidation potential. In summary, the connections between alternating dry–wet conditions (or water level fluctuations) and soil methanotrophic communities—specifically regarding their ecological adaptation and community dynamics, the development of community structure, and variations in community activity—remain insufficiently characterized.

### 4.3. Difference Between Oxygen Tolerance and Tolerance and Their Influence, as Well as the Role of Oxidation Micro-Zones

The hydrological fluctuations caused by the alternation of dry and wet conditions have a direct impact on the oxygen content of wetland soil samples. Wetland soils often present oxidized micro-patches at the millimeter to sub-millimeter scale, and their O_2_ concentration can be 2 to 3 orders of magnitude higher than that of the surrounding anaerobic substrate [72]. The physiological response of methane oxidizing bacteria (MOB) to O_2_ can be divided into two strategies: “tolerance” and “resistance.” The former refers to the ability of the bacteria to maintain the transcriptional level of pmoA under hypoxic conditions, while the latter emphasizes that the bacterial cells maintain enzymatic activity without oxidative damage even under high oxygen levels or atmospheric oxygen concentrations exceeding 21% [73]. Although methanotrophs are typically considered obligate aerobes requiring oxygen for growth, certain groups, such as those within the class Gammaproteobacteria (order Methylococcales), remain active under hypoxic conditions [41,74]. Under low-oxygen cultivation, gammaproteobacterial methanotrophs exhibit reduced biomass accumulation and release methane-derived organic compounds [75].

In the permanently flooded area, despite the extremely low REDOX potential (−200 to −350 mV), the highest methane oxidation potential was measured, which cannot be explained by traditional aerobic methane oxidation. Taniya Roy Chowdhury et al. speculated that this might be contributed by low-oxygen-tolerant Type II methano-oxidizing bacteria or anaerobic methano-oxidizing bacteria [33]. The “tolerance” or “adaptation” ability of such microorganisms to low-oxygen environments is key. Through metagenomic analysis, it was found that Methanosarcinales has repair system genes that are more adapted to oxidation, while Syntrophobacterales and Candidatus Mlm. oxyfera have VBNC marker genes. Candidatus Mlm. oxyfera is therefore an anaerobe that performs “intracellular aerobic methane oxidation”. It lives at the hypoxic/aerobic interface and uses the trace amount of intracellular O_2_ produced by its own metabolism to oxidize methane [76]. This indicates that oxidation micro-regions can even be created by the microorganisms themselves and are part of their oxygen tolerance strategy.

Moreover, methanotrophs utilize gas vesicles to navigate hypoxic environments. Composed of proteins, these vesicles not only provide buoyancy to aquatic organisms [77], allowing methanotrophs in anoxic zones to adjust their vertical position, but also increase intracellular contact area, optimizing the efficiency of methane oxidation under low-oxygen conditions. Gas vesicles have been identified in distinct lineages of aerobic methanotrophs under different environmental conditions. For instance, a psychrophilic Methylococcus-like strain (*Gammaproteobacteria*), isolated from permafrost, was reported to possess gas vesicles [78]. Research on aerobic methanotrophs oxidizing methane under oxygen-limited conditions is essential for predicting their contribution to the net methane balance in the context of future hydrological variability.

### 4.4. Influence of the Combined Effect of Temperature and Humidity

The interaction between temperature and moisture exerts a nonlinear synergistic effect on CH_4_-related processes, which is strongly modulated by seasonal drivers. Methanotrophs can enhance substrate availability under favorable conditions, contrasting with the accelerated loss of methane from wetland water columns due to ebullition during summer.

The interactive effect of temperature and moisture on CH_4_-related processes manifests as a nonlinear enhancement. The control experiment results reported by Deng et al. (2019) [79] demonstrated a significant increase in methane production as the temperature rose from 10 °C to 30 °C, whereas methane generation was nearly negligible at 45 °C. Methanogenic bacterial communities vary considerably across different soil types, and rising temperatures can induce shifts in the dominant microbial populations within the soil. These results indicated that the “temperature → moisture → redox → functional gene” cascade was the dominant mechanism. Several studies have demonstrated that extreme high temperatures exhibit a stronger correlation with precipitation and the global methane emission rate (WME), whereas extreme low temperatures are associated with a weaker correlation. However, when daily precipitation exceeds 10 mm, it initially enhances the WME, but excessive precipitation subsequently leads to a reduction in the emission rate [80]. The interaction between temperature and moisture (soil moisture) was the primary driving force for the spatio-temporal variation of CH_4_ flux in global wetlands, and its relative contribution far exceeded the single effect of temperature or moisture. This analysis will help us study and control methane emissions. Wang Yihui et al. (2024) [81] used a CLM-Microbe model to predict the methane emission of the Arctic tundra ecosystem, showing that the strong dependence of temperature and precipitation leads to the increase of methane emission, The positive feedback strengthening of the model will help us to improve the prediction of methane emission.

On the other hand, the synergistic effects of temperature and moisture are also reflected in the composition of methanotrophic communities. Under warm and humid conditions (e.g., tropical swamps, rice paddies), fast-growing type I methanotrophs (e.g., *Methylomonas*, *Methylococcus*), which are adapted to high methane concentrations, tend to dominate [82]. High temperatures and metabolic demands are matched by ample moisture and substrate supply [83]. In contrast, under cold and dry conditions (e.g., wetlands in cold regions, dry seasons), taxa with stronger environmental resilience prevail. Type II methanotrophs (e.g., *Methylosinus*, *Methylocystis*), which can form dormant cysts, may have a competitive advantage under low temperatures and fluctuating substrate availability [77]. In some constructed wetlands, species less affected by low temperatures exhibit greater stability and contribute more significantly to methane oxidation under cold conditions [79]. In fluctuating environments (e.g., alternating wet–dry or freeze–thaw zones) [80,81], the combined fluctuations are selected for strains with high physiological plasticity or rapid responsiveness. In such settings, communities may display elevated functional redundancy or be led by taxa characterized by rapid regulatory capacities in enzyme expression and osmotic balance.

## 5. Future CH_4_ Management in a Changing Climate

As climate change intensifies the hydrological cycle, the “hotspot” emissions of methane from wetlands present both risks and opportunities. Artificial hydrological scheduling serves as the most direct and effective lever for controlling CH_4_ emissions. Degraded wetlands will peak greenhouse gas emissions. It is estimated that by 2100, wetlands may produce greenhouse gas emissions equivalent to 408 tons [82], which will seriously affect our climate. Anthropogenic hydrological dispatching is the most direct and effective lever for controlling methane emissions.

A globally distributed Methylobacter clade 2 member, known as the dominant methane oxidizer in Arctic and Antarctic wetland soils, was recently successfully cultured from a tropical rice field ecosystem in India, bridging high-latitude and tropical methane cycling research. As shown in Table 3, the systematic analysis of methanogenic communities across different habitats enables the isolation of key taxa influencing methane emissions. This work paves the way for the targeted development of biological inhibitors to mitigate methane release.

These new strains successfully cultivated from tropical wetlands not only enrich our understanding of the global biodiversity of methane-oxidizing bacteria, but also provide key research materials for understanding their methane reduction function in tropical ecosystems, especially in the important artificial wetland of rice fields.

## 6. Conclusions

This review underscores that wetland methane emissions are far more than a straightforward biogeochemical flux; they emerge from an intricate interplay in which hydrological rhythms—specifically the magnitude, frequency and duration of wet–dry cycles—act as the dominant control, modulated by a suite of cross-scale factors.

At the microscale, oscillating water tables continually re-set soil redox potential (Eh) and oxygen diffusion, thereby tipping the balance between methanogenic archaea and methanotrophic bacteria and determining the net difference between methane production and oxidation.

At the macroscale, long-term hydrological regimes imprint functional traits on wetland vegetation (e.g., C/N ratio, aerenchyma development) and on soil physico-chemical properties, generating distinct emission signatures among tundra peatlands, boreal fens, mangroves, and other wetland types.

Consequently, understanding and forecasting wetland CH_4_ fluxes has moved beyond classical biogeochemistry and now demands convergence across microbial ecology, hydro-geomorphology, remote sensing, and ecosystem modelling.

Priority avenues for future research are as follows:

1. Mechanistic resolution of how key functional genes (pMMO, mcrA) respond to wet–dry alternations across minute-to-seasonal timescales.
2. A distributed, multi-scale in-situ network—such as the proposed Global Wetland Methane Observatory (GDWN)—to capture spatial and temporal heterogeneity worldwide.
3. Next-generation models that explicitly couple hydrological modules with microbial functional traits.

Only through such integrative efforts can we accurately quantify and predict the role of wetlands in the global methane budget under increasingly intense hydrological regimes, and provide the scientific foundation for nature-based management of these critical ecosystems.

## Figures and Tables

**Table 1:** Water level dynamics drive methane emission variability across ecosystems.

Ecosystem	Characteristics of Water Level Fluctuation	Magnitude of Methane Emissions	Key Controlling Factors
Floodplains	Natural, pulsed; large seasonal variation (ranging from meters to tens of meters).	Moderate emissions with high spatial heterogeneity.	Flood duration and the influx of fresh organic matter from flood pulses.
Mires	Relatively stable with high-frequency, low-amplitude fluctuations.	Consistently high methane source.	Permanently high water table, high organic matter content, abundant substrates for methanogens, and aerenchymatous plants (e.g., sedges).
Tundras	Unique dynamics dominated by seasonal freezing and thawing.	Currently low-to-moderate, but exhibits extremely high climate sensitivity.	Low temperatures limit microbial activity; climate warming threatens to unlock vast carbon stores, potentially transforming it into a strong source.
rice paddies	Intensively human-managed with drastic, regular fluctuations (oscillating between 0 cm and 5–10 cm during the growing season).	Moderate-to-high emissions with distinct seasonal peaks; manageable through practices.	Frequent alternate wetting and drying cycles, and the role of rice plants as conduits via aerenchyma.
reservoir drawdown zones	Artificially regulated, undergoing extreme seasonal changes (e.g., up to 30 m variation in the Three Gorges Reservoir).	Highly variable; acts as a “hotspot” for pulsed emissions.	Repeated anaerobic decomposition and aerobic mineralization of sediment organic matter due to drastic water level changes.

The summarized characteristics in this table are derived from the synthesis of the literature cited in the accompanying text.

**Table 2:** Kinetic parameters of methanotrophs under hydrological stress.

Parameters	Type I Methanotrophs	Type II Methanotrophs	ANME Consortia
CH_4_ affinity (Km)	1–5 μM	0.1–1 μM	10–50 μM
O_2_ optimum	15–21%	2–8%	<0.1%
Recovery post-drought	Slow (7–14 d)	Rapid (2–5 d)	>30 d

Data synthesized from Knief [68], Shiau et al. [61], and Valenzuela et al. [69].

**Table 3:** Summary of cultivated methanotrophic isolates from tropical wetland ecosystems.

Wetland Type	Specific Habitat Geographic Location	Isolated Methanotroph Species	Key Features/Strain Designation	Strain
Tropical Paddy Fields	Western Indian rice field soil	*Methylobacter* sp. KRF1 (Type I)	First cultured tropical member of globally important Methylobacter clade 2; contains molybdenum–iron and vanadium–iron nitrogenase genes	KRF1
	Western Indian rice field soil	*Methylocucumis oryzae* (Type I)	Large-sized, oblong-shaped cells forming pale pink colonies; isolated from stone quarries in wetland patches	Sn10-6
Tropical Freshwater Wetlands	Western Indian freshwater ponds and quarry lakes	*Methylomonas koyamae* (Type I)	Thermotolerant strain; originally described from rice fields, now isolated from wetland sediments	MgM2
	Western Indian freshwater ponds	*Methylosinus sporium* (Type II)	Purple non-sulfur methanotroph; forms cysts and rosettes	VLS4
Tropical Mangrove Wetlands	Mumbai and Alibag mangroves, India	*Methylocaldum gracile* (Type I)	Thermotolerant and halotolerant; dominates hot and humid coastal habitats	MgM4/MgD2/MgN2

Data synthesized from Rahalkar MC et al. [83,84] and Mohite JA et al. [85].

## Data Availability

No new data were created or analyzed in this study. Data sharing is not applicable.
